# A Comparison of Obstetric Outcomes in Antiphospholipid Syndrome Among Pregnant Women With Systemic Lupus Erythematosus

**DOI:** 10.7759/cureus.62126

**Published:** 2024-06-11

**Authors:** Dur e Shahwar, Duriya Rehmani, Amir Raza

**Affiliations:** 1 Obstetrics and Gynecology, Aga Khan University, Karachi, PAK

**Keywords:** intrauterine death, pregnancy, systemic lupus erythematosus, small for gestational age, preterm birth, eclampsia, preeclampsia, antiphospholipid syndrome

## Abstract

Objective

The aim of this study was to evaluate the maternal and perinatal outcomes in systemic lupus erythematosus (SLE) women with antiphospholipid syndrome (APS).

Methods

This retrospective case-control study was conducted among pregnant women with SLE with and without APS. Group A included SLE patients with APS, whereas group B included pregnant SLE women without APS. Data were expressed as mean ± standard deviation (SD). Frequency and percentage were computed for categorical data. The chi-square test was used to analyze the difference between categorical data.

Results

Out of 125 cases of SLE, APS was found in 72 (57.6%) women. Almost 95.8% of patients were on treatment (aspirin and enoxaparin) in group A. Preterm delivery (31.89±7.36 versus 34.46±4.97; p=0.021) and termination of pregnancy (18.1% [13/72] versus 5.7% [3/53]; p=0.04) were statistically significant in group A. Among these terminations, second-trimester intrauterine death is found to be more in group A (SLE with APS) (16.7% [12/72]) as compared to group B (SLE without APS) (5.7% [3/53]) with a p-value of 0.05. Perinatal outcomes including NICU admissions (39% [23/59] versus 24% [12/50]; p=0.071) and neonatal death (12.3% [7/57]; p=0.015) were also found to be statistically significant between the two groups.

Conclusion

APS with SLE is associated with adverse pregnancy outcomes such as preterm birth, termination of pregnancy due to second-trimester fetal loss, more NICU admission, and neonatal deaths when compared to the control group. Hence, pregnancies with APS with SLE require vigilant monitoring and frequent follow-ups to ensure a positive pregnancy outcome.

## Introduction

Antiphospholipid syndrome (APS) manifests as a condition marked by the existence of blood clotting issues and/or complications during pregnancy, all intertwined with the presence of certain antibodies known as antiphospholipid antibodies (aPLs). These antibodies, including lupus anticoagulant (LA), anticardiolipin antibodies, and anti-β2 glycoprotein-I antibodies (aβ2 GPI), serve as distinguishing hallmarks of this syndrome [1].

APS may exist alone in its primary form or may be in its secondary form where it is associated with other autoimmune diseases, particularly SLE [2]. The obstetric subset of antiphospholipid syndrome (OAPS) is characterized by the continuous presence of aPLs alongside specific obstetric complications. APS includes recurrent early pregnancy losses, fetal demise in the early stages of pregnancy, stillbirth, or premature birth before the 34th week of gestation due to conditions such as pre-eclampsia, eclampsia, or placental insufficiency [3].

APS exist without any symptoms in approximately 1% to 5% of women within the reproductive age bracket who have not experienced any prior instances of blood clot formation [4]. Approximately 20% to 40% of individuals with rheumatic diseases, including systemic lupus erythematosus (SLE), exhibit positivity for aPLs. Patients with SLE with positive aPLs, along with a history of thrombotic events, are at higher risk of complications during pregnancy [5]. Among these patients, high-titer IgG with positive LA has also been associated with an increased risk of pregnancy complications [6,7]. A meta-analysis conducted by do Prado et al. reported a positive relationship between anticardiolipin (aCL) antibodies and pre-eclampsia [8].

Zeisler has identified the expression of angiogenic biomarkers of placental insufficiency to predict and diagnose pre-eclampsia in high-risk population [9]. There are a lot of data that show that treatment with low-dose aspirin and low-molecular-weight heparin (LMWH) or unfractionated heparin has led to an increase in live births in 71% of APS pregnancies [10]. Women with SLE and APS should have pre-pregnancy counselling, and pregnancy should be planned with low disease activity before conception to ensure good pregnancy outcomes [11].

Rationale

The rationale of this study was to evaluate the practices for the management of APS among pregnant women with SLE and to analyze the obstetric outcomes. The results of this study would assist obstetricians and rheumatologists in counseling pregnant women with SLE and APS.

## Materials and methods

This is a retrospective case-control study of 125 pregnant patients with SLE, who delivered from January 1998 to December 2019 at the Aga Khan University Hospital. All SLE patients met the American College of Rheumatology (ACR) 1997 classification criteria [12]. The diagnosis of APS was made according to the revised International Classification Criteria established in 2006, which are based on both clinical manifestations and laboratory findings to reduce the possibility of selection bias. A total of 125 pregnant women with SLE who delivered during this period were included in the study using ICD-9 coding and were divided into two groups: group A included pregnant SLE patients with APS, and group B included pregnant women with SLE without APS. Majority of patients with APS received treatment with aspirin and enoxaparin. Pregnant women with a lack of complete medical information regarding disease were excluded.

The identification of SLE flareup was recorded according to the presence of new onset of anemia, lymphocytopenia, rising anti-ds DNA titer, declining C3 and C4 levels, or mark of increased proteinuria in the absence of pre-eclampsia.

A medical charts review was conducted for anticardiolipin IgG, IgM, LA, antibodies to Sm, Ro/SS-A, and La/SS-B, whereas SLE disease activity was monitored by levels of anti-dsDNA, C3, and C4 in each trimester. The demographic variables included age, weight, BMI, parity, gestational age at booking, and gestational age at delivery. The pregnancy outcomes were measured as previous history of miscarriages, pre-eclampsia, eclampsia, thrombocytopenia, intrauterine death (IUD), preterm birth, vaginal deliveries, cesarean section, flareup of disease, maternal mortality, and treatment received for SLE and APS. The perinatal outcomes were small for gestational age (SGA), IUGR, Apgar score at 1 and 5 minutes, stay in NICU, and neonatal death. All the data were retrieved through hospital medical records and reviewed and analyzed.

Operational definitions

Positive anticardiolipin IgG, with anticardiolipin IgG level of more than 40 GPL before or during the index pregnancy, and/or positive anticardiolipin IgM, with anticardiolipin IgM level of more than 40 MPL before or during the index pregnancy, and/or positive LA (diluted Russell viper venom time screening to confirm ratio as more than 1.60 and remaining positive for two occasions at least 12 weeks apart) [13].

Statistical analysis

Statistical analyses were performed using Statistical Package for Social Sciences (SPSS) Version 19 (IBM Corp., Armonk, NY, USA). Data were expressed as mean ± standard deviation (SD) and analyzed using the independent sample t-test or Mann-Whitney U test. Frequency and percentage were computed for categorical data. The chi-square test or Fisher’s exact test was used to analyze the difference between categorical data. Statistical significance was accepted as p < 0.05.

## Results

There were 72 patients with APS among 125 pregnant women with SLE. A p-value of <0.05 was considered statistically significant. The average age of the patient was 30.05±4.16 years (range: 18-43 years). Of the 125 women, 45 (36%) were primiparous, 31 (24.8%) were multiparous, and 49 (39.2%) were nulliparous. Baseline characteristics of the SLE patients with and without APS are shown in Table 1. The mean gestational age at delivery was significantly different between the groups; the mean gestational age at delivery in groups A and B was 31.89±7.36 and 34.46±4.97 (p = 0.021), respectively. The history of miscarriages was clinically significant in group A, 23 (31.9%), as compared to group B, 12 (22.6%) (p=0.25). No other significant difference was observed in other baseline characteristics between the two groups (Table 1).

**Table 1:** Baseline characteristics of women (n=125) Results are expressed as mean ± standard deviation and n (%). Independent sample t-test was used for mean, and the chi-square test was used for proportion differences. APS, antiphospholipid syndrome; SLE, systemic lupus erythematosus

Variables	SLE with APS (group A), n=72	SLE without APS (group B), n=53	p-Value
Age at conception (years)	29.86±4.05	30.30±4.38	0.560
Weight (kg)	157.64±5.25	156.81±6.72	0.441
Height (cm)	59.15±11.36	60.58±10.16	0.469
BMI (kg/m^2^)	23.75±4.01	24.69±4.18	0.202
Gestational age at booking (weeks)	14.35±7.51	15.84±7.28	0.280
Gestational age at delivery (weeks)	31.89±7.36	34.46±4.97	0.021
Miscarriages	23 (31.9%)	12 (22.6%)	0.252
Parity			0.737
Nulliparous	29 (40.3%)	20 (37.7%)	
Primiparous	27 (37.5%)	18 (34%)	
Multiparous	16 (22.2%)	15 (28.3%)	

**Figure 1:**
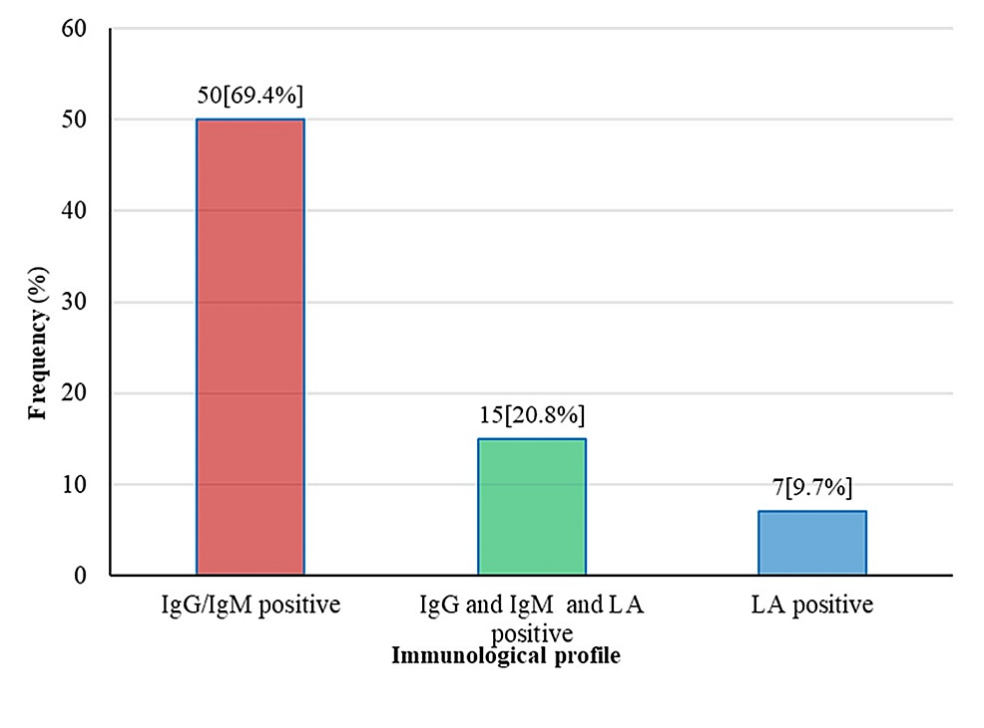
SLE patients with APS (n=72) APS, antiphospholipid syndrome; IgG, immunoglobulin A; IgM, immunoglobulin M; LA, lupus anticoagulant; SLE, systemic lupus erythematosus

Out of 125 cases, APS was found in 72 (57.6%) women. Anticardiolipin antibodies (IgG or IgM) were positive in 50 (69.4%) patients, anticardiolipin antibodies and LA were positive in 15 (20.8%) patients, and only LA was positive in seven (9.7%) SLE patients, as shown in Figure 1.

The overall SLE flareup was high in group A (SLE with APS). Nearly 25% of patients in group A conceived in the active phase of SLE, and 75% were in the remission phase at the time of conception. During pregnancy, SLE flareup occurred in 53 patients: 34 (64.2%) in the pre-conception phase, 18 (34%) in the antenatal phase, and 1 (1.9%) in the postnatal phase. Pre-conception flareup was clinically significant in group A, 64.2% (34/53), as compared to group B, 19 (57.6%). Among these patients, flareup occurred over a year ago before conception. During pregnancy, SLE flareups occurred in 23% (29/125) of patients; it was found more in group A, 18 (34%), as compared to group B, 11 (33.3%). In contrast, postnatal flareup was clinically significant among group B, 3 (9.1%). These results were clinically significant but not statistically significant, as shown in Table 2. 

**Table 2:** Comparison of clinical profile in SLE women with and without APS ANA, antinuclear antibodies; APS, antiphospholipid syndrome; SLE, systemic lupus erythematosus

Variables	SLE with APS (group A), n=72	SLE without APS (group B), n=53	p-Value
Disease activity at conception/booking visit			0.578
Active	18 (25%)	11 (20.8%)	
Remission phase	54 (75%)	42 (79.2%)	
Disease flareup	53 (73.6%)	33 (62.3%)	0.176
Time of flareup (n=86)	53	33	0.299
Preconceptional	34 (64.2%)	19 (57.6%)	
Antenatal	18 (34%)	11 (33.3%)	
Postnatal	1 (1.9%)	3 (9.1%)	
Positive ANA	61 (84.7%)	42 (79.2%)	0.427
Anti-DNA positive	59 (81.9%)	41 (77.4%)	0.526
C3 level <0.8	16 (22.2%)	11 (20.8%)	0.844
C4 level <0.1	14 (19.4%)	9 (17%)	0.725
Treatment received			
Aspirin	69 (95.8%)	0 (0%)	0.0005
Enoxaparin	69 (95.8%)	0 (0%)	0.0005
Hydroxychloroquine	55 (76.4%)	36 (67.9%)	0.293
Steroids	46 (63.9%)	39 (73.6%)	0.251
Azathioprine	22 (30.6%)	18 (34%)	0.687

Similarly, immunological markers including positive ANA, anti-DNA, C3 level < 0.8, and C4 < 0.1 were also high in group A, but no statistically significant difference was observed between the two groups (Table 2). In terms of treatment, most of the patients in group A were treated with aspirin and LMWH, 95.8% (69/72), because of a strong association of SLE with APS. Only three patients did not receive antithrombotic prophylaxis throughout pregnancy and stopped at 24 weeks of gestation due to financial limitations. The frequency of treatment with hydroxychloroquine, steroids, and azathioprine has been described in Table 2.

Fetomaternal outcomes were also found to be statistically significant in group A as compared to group B. Maternal outcomes included more patients with pre-eclampsia in group A, 29.2% (21/72), versus group B, 28.3% (15/53), eclampsia in group A, 5.6% (4/72), and an increase rate of cesarean section in group A, (59.7% 43/72), as compared to group B, 54.7% (29/53), though these results were not statistically significant between two groups. Maternal mortality was also higher in group A, 4 (5.6%), as compared to group B, 2 (3.8%) (p=0.645). There was a significant increase in the rate of termination of pregnancy due to fetal loss in group A (18.1%, 13/72) as compared to group B (5.7%, 3/53) (p=0.04). Second-trimester IUD was also found to be statistically significant in group A (16.7%, 12/72) as compared to group B (5.7%, 3/53). The perinatal outcomes including SGA fetus were more in group A (25.4%) than group B (14%), similarly growth restricted fetuses were also high (28.8%) in group A as compared to group B (26%), which was clinically significant between the two groups. Other neonatal outcomes including neonatal death, 12.3% (7/57) versus 0, and NICU admissions, 39% (23/59) versus 24% (12/50), were found to be statistically significant between the two groups (Table 3).

**Table 3:** Feto-maternal outcomes in SLE women with and without APS ^a^Chi-square test of association; p-value less than 0.05 was taken as significant. ^b^Denominator changed due to termination of pregnancy. APS, antiphospholipid syndrome; IUD, intrauterine death; IUGR, intrauterine growth restriction; NICU, neonatal intensive care unit; SGA, small for gestational age; SLE, systemic lupus erythematosus

Maternal outcomes	SLE with APS (group A), n=72	SLE without APS (group B), n=53	Total (n=125)	p-Value^a^
Pre-eclampsia	21 (29.2%)	15 (28.3%)	36 (28.8%)	0.916
Eclampsia	4 (5.6%)	0	4 (3.2%)	0.136
Thrombocytopenia	5 (6.9%)	7 (13.2%)	12 (9.6%)	0.240
Maternal mortality	4 (5.6%)	2 (3.8%)	6 (4.8%)	0.645
Mode of delivery				
Cesarean section	43 (59.7%)	29 (54.7%)	72 (57.6%)	0.234
Vacuum/forceps	0 (0%)	2 (3.8%)	2 (1.6%)	
SVD	16 (22.2%)	19 (35.8%)	35 (28%)	
Termination of pregnancy	13 (18.1%)	3 (5.7%)	16 (12.8%)	0.040
IUD	15 (20.8%)	6 (11.3%)	21 (16.8%)	0.160
Second trimester	12 (16.7%)	3 (5.7%)	15 (12%)	0.052
Third trimester	3 (4.2%)	3 (5.7%)	6 (4.8%)	0.698
Preterm birth				
≤34 weeks birth^b^	14/59 (23.7%)	10/50 (20%)	24/109 (22%)	0.640
<37 weeks birth^b^	46/59 (78%)	33/50 (66%)	79/109 (72.5)	0..163
Fetal and neonatal outcomes				
Percentile				
SGA (<10 percentile)	15/59 (25.4%)	7/50 (14%)	22/109 (20.2%)	0.139
IUGR	17/59 (28.8%)	13/50 (26%)	30/109 (27.5%)	0.743
Apgar score				
<7 at 1 minute	5/59 (8.5%)	2/50 (4%)	7/109 (6.4%)	0.449
<7 at 5 minutes	0	0	0	NA
NICU admissions	23/59 (39%)	12/50 (24%)	35/109 (32.1%)	0.071
Neonatal death	7/57 (12.3%)	0 (0%)	7/104 (6.7%)	0.015

There were no significant differences between groups A and B in the rates of preterm births at less than 34 weeks of gestation, 23.7% (14/59) versus 20% (10/50), and Apgar score of less than 7 at 1 minute, 8.5% (5/59) versus 4% (2/50) (Table 3).

Subgroup analysis was also conducted, which showed that IUD and Apgar score less than 7 at 1 minute were considerably higher in women who had both positive anticardiolipin antibodies and positive LA, whereas neonatal deaths were more in patients with positive anticardiolipin antibodies, as shown in Table 4. 

**Table 4:** Comparison of outcome: Subgroups of SLE patients with APS and SLE patients without APS ^a^Chi-square test of association, p-value less than 0.05 taken as significant. ANA, antinuclear antibodies; APS, antiphospholipid syndrome; IUD, intrauterine death; IUGR, intrauterine growth restriction; NICU, neonatal intensive care unit; SGA, small for gestational age; SLE, systemic lupus erythematosus

Variables	Subgroup of SLE patients with APS			SLE without APS, n=53	p-Value^a^
	Positive IgG/IgM, n=50	IgG/IgM >3% and positive LA, n=15	Positive LA, n=7		
Disease flareup	38 (76%)	11 (73.3%)	4 (57.1%)	33 (62.3%)	0.416
Disease activity at conception					
Active	12 (24%)	3 (20%)	3 (42.9%)	11 (20.8%)	0.615
Remission phase	38 (76%)	12 (80%)	4 (57.1%)	42 (79.2%)	
Thrombocytopenia	3 (6%)	1 (6.7%)	1 (14.3%)	7 (13.2%)	0.600
Positive ANA	41 (82%)	15 (100%)	5 (71.4%)	42 (79.2%)	0.245
Anti-DNA positive	39 (78%)	14 (93.3%)	6 (85.7%)	41 (77.4%)	0.539
C3 level <0.8	11 (22%)	2 (13.3%)	3 (42.9%)	11 (20.8%)	0.475
C4 level <0.1	9 (18%)	2 (13.3%)	3 (42.9%)	9 (17%)	0.373
Cesarean section	33/44 (75%)	5/10 (50%)	5/5 (100%)	29/50 (58%)	0.081
Pre-eclampsia	13 (26%)	4 (26.7%)	4 (57.1%)	15 (28.3%)	0.396
Eclampsia	3 (6%)	1 (6.7%)	0	0	0.280
Maternal mortality	2 (4%)	1 (6.7%)	1 (14.3%)	2 (3.8%)	0.640
IUD	7 (14%)	6 (40%)	2 (28.6%)	6 (11.3%)	0.048
Preterm ≤34 weeks	9/44 (50.5%)	3/10 (30%)	2/5 (40%)	10/50 (20%)	0.684
Preterm <37 weeks	34/44 (77.3%)	8/10 (80%)	4/5 (80%)	33/50 (60%)	0.576
Centile					
<10	10/43 (23.3%)	4/9 (44.4%)	1/5 (20%)	7/47 (14.9%)	0.246
IUGR	8/43 (18.6%)	2/9 (22.2%)	0	4/47 (8.5%)	0.343
Apgar score <7 at 1 minutes	2/43 (4.7%)	3/9 (33.3%)	0	2/47 (4.3%)	0.010
NICU admissions	19/43 (44.2%)	3/9 (33.3%)	1/5 (20%)	12/47 (25.5%)	0.268
Neonatal death	6/43 (14%)	1/9 (11.1%)	0	0	0.055

## Discussion

This study aims to elucidate the specific obstetric outcomes and associated risk factors among SLE patients with APS, contributing valuable insights to the management and care of this high-risk patient population. Baseline characteristics of women among SLE patients with and without APS is illustrated in Table 1.

Our data have shown differences in gestational age at the time of delivery in both groups. The mean gestational age at the time of delivery in SLE patients with APS was significantly lower than the SLE patients without APS, with a p-value of 0.021, which was statistically significant. Our findings are comparable to the study that shows high preterm delivery rates in APS due to pregnancy complications [14]. The difference was observed in miscarriage rates in patients between group A Group B, and it was clinically significant as illustrated in Table 1. It is in accordance with a study by Schreiber and Hunt, which also shows that antiphospholipid antibodies (aPLs) directly impact the development of the placenta and cause programmed cell death (apoptosis) in trophoblast cells. This influence could be significant, especially in cases of repeated early pregnancy losses [15].

Table 2 illustrates the comparison among SLE patients based on their aPL status. In our study, higher numbers of periconceptional flareups were observed more in group A than group B, but that flareup occurred more than one year before conception, which showed that more SLE patients with APS (group A) conceived in the remission phase of disease as compared to group B. These results are identical to those reported in the PROMISSE (Predictors of Pregnancy Outcome in Systemic Lupus Erythematosus and Antiphospholipid Syndrome) study, which is so far the largest multicenter cohort study on lupus and has shown the presence of LA as a strong predictor of adverse pregnancy outcomes and increased flareups in pregnancy. In the PROMISSE trial, the cohort flare rates in the second and third trimesters were 2.5% and 3%, respectively [16]. These figures contradict our study finding, which shows much higher rates of antenatal flareups in groups A and B.

Table 2 also illustrates the medical treatment received by the patients. Aspirin and enoxaparin were the first-line treatment given to SLE patients with APS. However, aspirin and enoxaparin were not prescribed to any patient with SLE in the absence of APS. Almost 95.8% of patients in group A received aspirin and enoxaparin. Capecchi et al. in a systematic review concluded that patients with obstetric APS should receive heparin and low-dose aspirin throughout pregnancy to prevent adverse pregnancy outcomes [17]. A Cochrane review has also evaluated the role of aspirin and heparin in improving pregnancy outcomes [18].

Table 3 shows a comparison of maternal and fetal outcomes in both the groups. The maternal outcomes include pre-eclampsia and eclampsia, both of which were comparable between both groups. There was no patient with eclampsia in group B. The answer lies in the fact that almost all the patients with APS had received aspirin and enoxaparin from the start of pregnancy. A recent study on APS treatment has also suggested the use of aspirin and heparin in obstetric APS in preventing complications [19].

Data from different studies have shown higher mortality in patients with APS. Dabit et al. have concluded that mortality in APS is 50% to 80% higher than in the general population [20]. Our study has also shown higher mortality in patients with APS (group A). There were four (5.6%) maternal deaths in group A and two (3.8%) in group B. This difference was clinically significant. In group A, all the patients had lupus nephritis with APS, and the main cause of death was sepsis and multiorgan failure. A systemic review by Ritchie et al. also illustrated that the major causes of death in patients with SLE was active disease and sepsis [21]. This finding is similar to our study in which we also observed these two factors as the major cause of maternal deaths in the groups.

The difference in intrauterine deaths between groups A and B is shown in Table 3. In group A, 16.7% of IUDs were in the second trimester and 4.2% in the first trimester, the peak occurrence of fetal loss was between 12 and 23 weeks of gestation, and two stillbirths occurred in the third trimester (from 32 to 36 weeks of gestation) in patients with positive LA. These findings are similar to other studies which have shown a high perinatal loss rate in patients with APS. Singh and Sidhu have evaluated APS as a cause of bad obstetric outcomes [22]. There were more babies with growth percentile <10 in group A than group B, which was clinically significant. The prevalence of IUGR was more or less comparable between the two groups, as shown in Table 3. These findings correspond to the study by Abou-Nassar et al., which has shown that APS is associated with pre-eclampsia and IUGR [23].

Preterm birth rates are higher in group A, as shown in Table 3, and the mean reason for NICU admission. Neonatal mortality was also found to be higher in group A as compared to group B, which is similar to the study conducted by Fazzari et al. [24].

Table 4 illustrates the subgroup analysis of APS among SLE patients. Group one has only IgG and IgM positive, second group has IgG, IgM, and LA positive, and the third group has only LA-positive antibodies. Maternal and fetal outcomes are compared among these three subgroups. Pre-eclampsia, maternal mortality, and cesarean section rates were clinically significant in LA-positive group among APS patients. By further subdividing the data with APS into three subgroups, we have observed that the highest perinatal mortality rates and preterm births both were seen more in patients with both APS (both IgG/IgM positive) and LA. These findings correspond to a meta-analysis conducted by Xu et al., who concluded that LA is strongly associated with late fetal loss in APS patients [25].

Our study has shown that the subset of APS with LA is associated with the highest number of intrauterine deaths, which corresponds to the findings of the PROMISSE study, which also concluded the same.

Limitations

The limitation of this study is that although it is conducted in a tertiary care center, it is unicentral and retrospective in nature with a small sample size. It includes the long-term data of 20 years, which highlighted numerous important predictors of poor pregnancy outcomes in SLE patients with APS,but results of the study cannot be generalized and we need more prospective, multicenter studies to generalize the results.

Strength of the study

One of the strengths of this study is that it was conducted in one of the largest tertiary care centers of the country, with a large number of referrals from different ethnic groups; therefore, it truly reflects the burden of APS in the obstetric population and their outcomes.

## Conclusions

APS along with SLE is associated with adverse pregnancy outcomes such as preterm birth, second-trimester fetal loss, NICU admission, and neonatal deaths when compared to SLE alone. Hence, pregnancy with APS with concomitant SLE require frequent follow-ups and vigilant monitoring throughout pregnancy to ensure a positive maternal and fetal outcome.

## References

[1] Ruffatti A, Salvan E, Del Ross T (2014). Treatment strategies and pregnancy outcomes in antiphospholipid syndrome patients with thrombosis and triple antiphospholipid positivity. A European multicentre retrospective study. Thromb Haemost.

[2] Tektonidou MG, Andreoli L, Limper M (2019). EULAR recommendations for the management of antiphospholipid syndrome in adults. Ann Rheum Dis.

[3] Erton ZB, Sevim E, de Jesús GR (2022). Pregnancy outcomes in antiphospholipid antibody positive patients: prospective results from the AntiPhospholipid Syndrome Alliance for Clinical Trials and InternatiOnal Networking (APS ACTION) Clinical Database and Repository ('Registry'). Lupus Sci Med.

[4] Andreoli L, Chighizola CB, Banzato A, Pons-Estel GJ, Ramire de Jesus G, Erkan D (2013). Estimated frequency of antiphospholipid antibodies in patients with pregnancy morbidity, stroke, myocardial infarction, and deep vein thrombosis: a critical review of the literature. Arthritis Care Res (Hoboken).

[5] Ünlü O, Zuily S, Erkan D (2016). The clinical significance of antiphospholipid antibodies in systemic lupus erythematosus. Eur J Rheumatol.

[6] Clowse ME, Magder LS, Witter F (2006). Early risk factors for pregnancy loss in lupus. Obstet Gynecol.

[7] Buyon JP, Kim MY, Guerra MM (2015). Predictors of pregnancy outcomes in patients with lupus: a cohort study. Ann Intern Med.

[8] Do Prado AD, Piovesan DM, Staub HL (2010). Association of anticardiolipin antibodies with preeclampsia: a systematic review and meta-analysis. Obstet Gynecol.

[9] Zeisler H, Llurba E, Chantraine F (2016). Predictive value of the sFlt-1:PlGF ratio in women with suspected preeclampsia. N Engl J Med.

[10] Clark EA, Silver RM, Branch DW (2007). Do antiphospholipid antibodies cause preeclampsia and HELLP syndrome?. Curr Rheumatol Rep.

[11] Yamada H, Atsumi T, Kobashi G (2009). Antiphospholipid antibodies increase the risk of pregnancy-induced hypertension and adverse pregnancy outcomes. J Reprod Immunol.

[12] Hochberg MC (1997). Updating the American College of Rheumatology revised criteria for the classification of systemic lupus erythematosus. Arthritis Rheum.

[13] Miyakis S, Lockshin MD, Atsumi T (2006). International consensus statement on an update of the classification criteria for definite antiphospholipid syndrome (APS). J Thromb Haemost.

[14] Clark CA, Spitzer KA, Laskin CA (2005). Decrease in pregnancy loss rates in patients with systemic lupus erythematosus over a 40-year period. J Rheumatol.

[15] Schreiber K, Hunt BJ Managing antiphospholipid syndrome in pregnancy. Thromb Res. 2019 Sep.

[16] Yelnik CM, Laskin CA, Porter TF (2016). Lupus anticoagulant is the main predictor of adverse pregnancy outcomes in aPL-positive patients: validation of PROMISSE study results. Lupus Sci Med.

[17] Capecchi M, Abbattista M, Ciavarella A, Uhr M, Novembrino C, Martinelli I (2022). Anticoagulant therapy in patients with antiphospholipid syndrome. J Clin Med.

[18] Hamulyák EN, Scheres LJ, Marijnen MC, Goddijn M, Middeldorp S (2020). Aspirin or heparin or both for improving pregnancy outcomes in women with persistent antiphospholipid antibodies and recurrent pregnancy loss. Cochrane Database Syst Rev.

[19] Grygiel-Górniak B, Mazurkiewicz Ł (2023). Positive antiphospholipid antibodies: observation or treatment?. J Thromb Thrombolysis.

[20] Dabit JY, Valenzuela-Almada MO, Vallejo-Ramos S, Duarte-García A (2022). Epidemiology of antiphospholipid syndrome in the general population. Curr Rheumatol Rep.

[21] Ritchie J, Smyth A, Tower C, Helbert M, Venning M, Garovic V (2012). Maternal deaths in women with lupus nephritis: a review of published evidence. Lupus.

[22] Singh G, Sidhu K (2010). Bad obstetric history: a prospective study. Med J Armed Forces India.

[23] Abou-Nassar K, Carrier M, Ramsay T, Rodger MA (2011). The association between antiphospholipid antibodies and placenta mediated complications: a systematic review and meta-analysis. Thromb Res.

[24] Fazzari MJ, Guerra MM, Salmon J, Kim MY (2022). Adverse pregnancy outcomes in women with systemic lupus erythematosus: can we improve predictions with machine learning?. Lupus Sci Med.

[25] Xu J, Chen D, Duan X, Li L, Tang Y, Peng B (2019). The association between antiphospholipid antibodies and late fetal loss: a systematic review and meta-analysis. Acta Obstet Gynecol Scand.

